# Editorial: New Approaches in Toxicity Testing of Nanotherapeutics

**DOI:** 10.3389/fphar.2022.922551

**Published:** 2022-06-14

**Authors:** Suresh K. Kalangi, Rajesh Bhosale

**Affiliations:** ^1^ Amity Stem Cell Institute, Amity Medical School, Amity University Gurugram, Amity Education Valley, Gurugram, India; ^2^ Department of Chemistry, School of Science, Indrashil University, Rajpur, India

**Keywords:** nanotoxicity, cancer imaging, nano drug delivery, quantum dot, aggregation induced emission (AIE), bio distribution

Disease intervention of a clinician involves two important facets—diagnosis and therapy. Irrespective of whether it is a systemic disease or infectious disease, diagnosis is a mandatory prerequisite. An interdisciplinary approach in detecting the early alterations in the host body and enhancing drug efficacy is an essential requirement. Consequentially, the challenges of effective therapeutic intervention are now being met by synthesizing efficient nonmaterial which is successful in enhancing bioavailability of the drug or by engineering sensor molecules to detect the early diagnostic changes (biomarkers) inside the body.

Over the last few decades, nanotechnology has emerged as the most promising avenue to address the challenging issues in the fields of modern science such as environmental, electronics, healthcare, and many more (Singh et al., 2019). In the area of modern medicine, nanotechnology is playing a pivotal role in ultrasensitive diagnosis of diseases, site specific/organ-oriented imaging, targeted nano-carrier drug delivery, targeted nano-based phototherapy, and tracking of drug delivery (Wong et al., 2020). Despite the huge potential applications in diagnosis and therapeutics, nanotechnology is also associated with adverse effects like nanomaterial deposition in the host body and issues of toxicity in healthy cells, tissues, and other organs (Al-Ahmady and Ali-Boucetta, 2020). To address these issues, nanotoxicology is emerging as a specialized field of research with the focus of assessing nanomaterial safety aspects on human health, a subject which is elusive and not yet understood in greater detail (Akçan et al., 2020). This Research Topic at Frontiers in Pharmacology aims to emphasize the current status of toxicity assays for nanotherapeutics and discuss the present need for developing novel approaches to test toxicity.

Among nanoparticle safety issues, neurotoxicity of nanoparticles has the least studies. A review article published by Vinod and Jena has debated that biosynthesized nanoparticles possessing biocompatible surface functional groups exhibit lower neural toxicity than chemically prepared nanoparticles. An interesting observation was that biosynthesized nanoparticles manifest toxicity upon disintegrating to their simpler metabolite forms. The toxic behavior of nanoparticles seems to be influenced by many aspects like size and surface area, capping agents used in surface coating, degree of crystallinity, dissolution in body fluids, and agglomeration. This review also ascertained the utilization of biodegradable nanoparticles for the application of nano-neurotheranostics . The toxicity exhibited by nanoparticles may be due to oxidative stress, reactive oxygen species (ROS), irreversible interruption of neurological functions, nanoparticles-based genotoxicity, and many others. Contemporary methods of testing toxicity of nanotherapeutics are highlighted in the published review article by Tirumala et al. This review has extensively discussed the generalized toxicity mechanisms induced by nanoparticles besides highlighting the conventional and novel noninvasive methods of toxicity assays availed to evaluate them. The cytotoxicity of nanoparticles can be measured by various assays such as 3-(4,5-dimethylthiazol-2-yl)-2,5-diphenyl tetrazolium bromide (MTT), 2,3-bis-(2-methoxy-4-nitro-5-sulfophenyl)-2H-tetrazolium-5-carboxanilide (XTT), neutral red dye, resazurin, trypan blue, propidium iodide, adverse outcome (AO) staining assays, terminal deoxynucleotidyl transferase (TdT) dUTP nick-end labeling (TUNEL) annexin-V, bromodeoxyuridine (BrdU) assays, and thymidine (3H-TdR). Genotoxicity can be evaluated by single-cell electrophoresis, cytokinesis-block micronucleus (CBMN), and chromosomal aberration assays. Immunotoxicity can be examined by enzyme-linked immunosorbent assay (ELISA) and RT-PCR methods. The level of ROS formation can be monitored by methods of glutathione (GSH), dichlorodihydrofluorescein diacetate (DCFDA), and a MitoSOX-based assay. Inflammation specific to nanoparticles can be observed by release of inflammatory mediators like nitric oxide inflammatory cytokines. Notwithstanding the above methods, computational models such as quantitative structure-activity relationship (QSAR) modeling, omics technologies, and green algorithms also play a key role in the evaluation of nanoparticle-based toxicity.

The contemporary understanding of various non-communicable diseases such as cancer, diabetes, etc., emphasizes the need for personalized medicine. It calls for the synergy of chemistry, material, and biology scientists to focus on the development of personalized medicine or point-of-care medicine to ensure a secure healthy future. Study progress in this direction is replacing time-consuming and expensive conventional drug discovery studies. These modern data-driven approaches can make the drug discovery pipeline shorter and cost-efficient. The shortcomings of successful drug testing on pre-clinical animal models enroute to human trials are challenging and demand a network of scientists across disciplines to work in consortium to develop more appropriate efficient disease models such as organoids or lab/organ-on-chip systems for pre-clinical drugs. These studies and testing models can accelerate the process of drugs reaching the market. In an era of no violence or reduced animal usage, predictive toxicology or alternative animal models will prove to be the perfect platform for novel drug discovery, especially in the case of nano formulations. In recent years, stem cells have emerged as a novel approach to predict the toxicity of nanoparticles. Modern advanced analytical methods such as lab-on-chip-based 3D micro/nano-fluidics, 3D liver bio-printing, and 3D organoid scaffolds expose organ toxicity *via* nanoparticles. Electrochemical-based bio-electrochemical methods can evaluate toxicity effects of nanoparticles (*in vitro* and *in vivo*) non-invasively at multicellular as well as unicellular levels.

With the recent entry of new nanotheranostic probes: natural (Lu et al., 2022) and chemically synthesized aggregation-induced emission (AIE)-based nanoparticles, there is a global impetus for the development of advanced methods based on AIE. AIE-based technologies can revolutionize fields like clinical diagnosis, imaging-guided photodynamic therapy, optoelectronics, and emission signaling for predicating various diseases (Chen et al., 2022). Further, AIE-based luminogens (AIEgens) have become an alternative to metal-based nanoparticles due to their significantly enhanced emission upon the formation of aggregates, excellent contrast imaging, and also avoidance of the aggregation-caused quenching (ACQ) effect (which limits the application of conventional fluorescent dyes/nanoparticles like quantum dots (QDs)). Though AIEgen materials are mostly biocompatible, a few recent reports suggested adverse immunological effects, highlighting the new dimension of nanomaterial toxicity to be studied in detail which may deviate from immunological adverse outcomes of metal-based nanoparticles (Wu et al., 2020). In the present advanced dynamically evolving era of novel nanotheranostics materials and applications, the patient-specific cell-based/organ-based aspect of nanotoxicity can only provide a partial overview of nanotoxicity. However, in-depth understanding of pathways involved in the failure of clearing the nanoparticle deposition at an organism level and *in vivo* biodistribution, the corresponding immunological effects, and key molecular interactions of AIE probes/nanodots with biological fluids need to be deciphered to provide better safe usage of nanotherapeutics/diagnostics in human healthcare. Overall, this Research Topic summarizes the existing, advanced, futuristic nanotoxicology approaches for assessing the safety of various nanoparticles and nanotheranostic probes.
